# Erucism due to cup moth

**DOI:** 10.11604/pamj.2018.30.16.15185

**Published:** 2018-05-08

**Authors:** Andreia de Oliveira Alves, Fred Bernardes Filho

**Affiliations:** 1 Medical School, Centro Universitário Barão de Mauá, Ribeirão Preto, São Paulo, Brazil; 2 Dermatology Division, Department of Medical Clinics, Ribeirão Preto Medical School, University of São Paulo, Ribeirão Preto, Brazil

**Keywords:** Lepidoptera, pruritus, urticaria

## Image in medicine

A 12-year-old girl was assessed because of a 1-hour history of severe pruritus after contact with a cup moth (A). She has past history of anaphylactic shock to bee sting two years ago. The patient was sitting next to a mango tree and inadvertently put her left arm over a caterpillar. Physical examination revealed an erythematous infiltrated plaque with moderate swelling and an urticarial eruption with some excoriations on left upper limb (B, C); patient had a pulse rate of 78/min, blood pressure of 108/64 mmHg and respiratory rate of 15/min. The patient received prednisone 40 mg/day and loratadine 10mg/day for 5 and 10 days, respectively. Two weeks after the accident, on the final follow-up visit, the patient showed no late effects. Erucism, also called caterpillar dermatitis, is caused by envenomation by the larval or pupal stages of moths or butterflies. Caterpillars transmit their venom through urticating hairs, spines, or setae distributed over their bodies. Rash is the most common clinical manifestation and can range from a mild itching to a very painful sting. The lesions are provoked by caterpillar bristles filled with toxins that penetrate the skin. The bristles are hollow and when they enter the skin and break, toxins that contain thermolabile proteins, proteolytic enzymes and histamine are released. Treatment is supportive and includes washing the site with soap and water, applying cold water compresses, and administering topical or oral antihistamines or corticosteroids.

**Figure 1:**
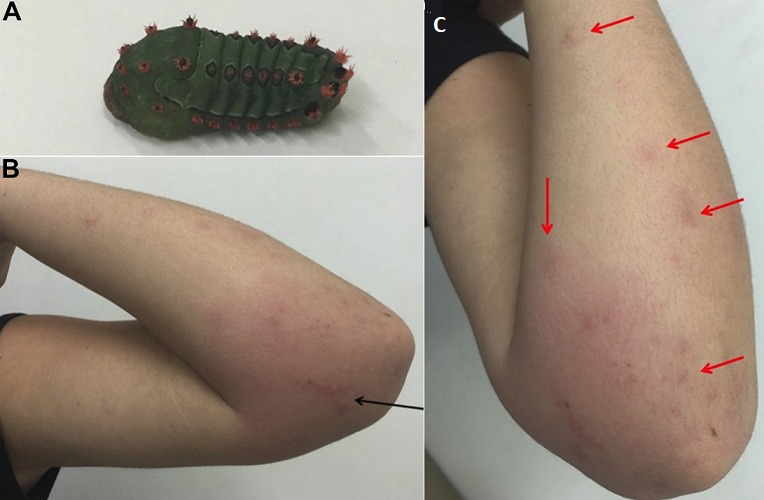
A) renamented and brightly colored caterpillar brought by the patient’s mother to the emergency room; (Lepidoptera: Limacodidae); B) erythematous infiltrated plaque with moderate swelling; a purplish area can be observed in the center of the lesion (arrow); C) multiple scattered erythematous papules with excoriations (red arrows), particularly on the extensor aspect

